# Efficacy of PD-1/PD-L1 inhibitors combined with multi-targeted anti-angiogenic TKIs in advanced or metastatic NSCLC: A meta-analysis based on RCTs

**DOI:** 10.3389/fonc.2026.1799126

**Published:** 2026-04-01

**Authors:** Zeqi Tang, Xiaoming Zhang, Zhanqi Sun, Shiyan Zhang, Xiaoling Feng, Yixia Chen, Haiqi Xu

**Affiliations:** 1 The Fifth School of Clinical Medical, Zhejiang Chinese Medical University, Hangzhou, Zhejiang, China; 2 The First Clinical Medical College, Zhejiang Chinese Medical University, Hangzhou, Zhejiang, China; 3 Health Science Center, Ningbo University, Ningbo, Zhejiang, China; 4 The Second Clinical Medical College, Zhejiang Chinese Medical University, Hangzhou, Zhejiang, China; 5 Department of Respiratory and Critical Care Medicine, Ningbo Hospital of Integrated Traditional Chinese and Western Medicine, Ningbo, Zhejiang, China

**Keywords:** meta-analysis, multi-targeted anti-angiogenic TKIs, NSCLC, os, PD-1/PD-L1 inhibitors, PFS

## Abstract

**Background:**

The efficacy of combining programmed cell death protein-1 (PD-1)/programmed death-ligand 1 (PD-L1) inhibitors with multi-targeted anti-angiogenic tyrosine kinase inhibitors (TKIs) in advanced or metastatic non-small cell lung cancer (NSCLC) remains controversial. This study therefore aimed to systematically evaluate the efficacy of this combination regimen in patients with advanced or metastatic NSCLC.

**Methods:**

We systematically searched PubMed, EMBASE, Web of Science, ClinicalTrials.gov, and the Cochrane Library databases for relevant randomized controlled trials (RCTs) up to July 2025. The primary outcomes were progression-free survival (PFS) and overall survival (OS), analyzed using the hazard ratio (HR) and 95% confidence interval (95%CI).

**Results:**

A total of six RCTs were included, involving 2,787 participants. Results demonstrated that the intervention group receiving PD-1/PD-L1 inhibitors combined with anti-angiogenic TKIs showed improved PFS compared with the control group (HR = 0.82, 95%CI: 0.69-0.97, p=0.021). However, no statistically significant difference was observed between the two groups in OS (HR = 0.97, 95%CI: 0.88-1.07, p=0.554). Subgroup analyses indicated that the PFS benefit was more pronounced in patients aged<65 years (HR = 0.78, 95%CI: 0.64-0.94), female (HR = 0.73, 95%CI: 0.57-0.92), never-smokers (HR = 0.72, 95%CI: 0.53-0.98), white people (HR = 0.85, 95%CI: 0.72-0.99), with non-squamous NSCLC (HR = 0.82, 95%CI: 0.66-1.01, p=0.064), high PD-L1 expression (tumor proportion score (TPS)≥50%) (HR = 0.78, 95%CI: 0.63-0.96), Eastern Cooperative Oncology Group (ECOG) performance status (PS)=1 (HR = 0.78, 95%CI: 0.64-0.96), no liver (HR = 0.78, 95%CI: 0.64-0.96) or brain (HR = 0.73, 95%CI: 0.53-1.00, p=0.054) metastases, and those receiving first-line therapy (HR = 0.72, 95%CI: 0.54-0.97). No significant difference in OS was observed in all subgroups (all p>0.05).

**Conclusion:**

The combination of PD-1/PD-L1 inhibitors and anti-angiogenic TKIs significantly improved PFS in patients with advanced or metastatic NSCLC but did not translate into an OS benefit.

**Systematic Review Registration:**

https://www.crd.york.ac.uk/PROSPERO/, identifier CRD420251126527.

## Introduction

Non-small cell lung cancer (NSCLC) is the most common histological subtype of lung cancer, accounting for approximately 85% of cases, with a five-year survival rate of only 26.4% (1). For advanced or metastatic NSCLC without actionable driver mutations, immune checkpoint inhibitors (ICIs), which mainly target the programmed cell death protein-1 (PD-1)/programmed death-ligand 1 (PD-L1), have become a cornerstone of first-line therapy. However, treatment efficacy remains unsatisfactory, primarily due to two key reasons. First, a significant proportion of patients derive limited benefit due to primary resistance (2). Second, even among initial responders, the long-term efficacy is often attenuated by the development of acquired resistance (2). Tumor resistance mechanisms are complex, involving tumor genomic aberrations, an immunosuppressive tumor microenvironment (TME), and altered tumor metabolism (2). Therefore, to enhance therapeutic efficacy, PD-1/PD-L1 inhibitors are frequently combined with other agents such as chemotherapy (3, 4), cytotoxic T-lymphocyte-associated protein 4 (CTLA-4) inhibitors (5), and anti-angiogenic drugs (6). Among these strategies, anti-angiogenic drugs—particularly multi-targeted anti-angiogenic tyrosine kinase inhibitors (TKIs)—have emerged as a distinct and highly promising therapeutic approach.

Multi-targeted anti-angiogenic TKIs, exemplified by lenvatinib, effectively inhibit tumor angiogenesis and progression by simultaneously blocking the phosphorylation of multiple key receptors—including vascular endothelial growth factor receptor (VEGFR), fibroblast growth factor receptor (FGFR), and platelet-derived growth factor receptor (PDGFR)—thereby suppressing downstream signal transduction (7). This multi-targeted property aids in overcoming signal crosstalk such as compensatory angiogenesis (8, 9). Further research indicates that lenvatinib, through its specific kinase inhibition profile, can alleviate immunosuppression in the TME via multiple mechanisms (10, 11). This dual regulation of the vascular system and immune microenvironment generates potent synergistic effects with PD-1/PD-L1 inhibitors, providing a theoretical basis for reversing immunosuppression and overcoming resistance. However, the combination of multi-targeted anti-angiogenic TKIs with PD-1/PD-L1 inhibitors remains controversial, primarily due to inconsistent efficacy between progression-free survival (PFS) and overall survival (OS) (12, 13).

To clarify this controversy, several meta-analyses have been conducted (14–16). Although previous meta-analyses have evaluated the efficacy of combining PD-1/PD-L1 inhibitors with anti-angiogenic therapy in advanced or metastatic NSCLC, they have typically combined two pharmacologically distinct classes of anti-angiogenic drugs—monoclonal antibodies and multi-targeted TKIs—into a single category. This approach has limited the ability to specifically assess the efficacy and value of TKIs. Therefore, this study aims to evaluate the efficacy of PD-1/PD-L1 inhibitors combined with multi-targeted anti-angiogenic TKIs in advanced or metastatic NSCLC through a meta-analysis, providing clinical reference.

## Methods

This meta-analysis was conducted and drafted in accordance with the standard guidelines and checklists of Preferred Reporting Items for Systematic Reviews and Meta-Analyses (PRISMA). Additionally, the study protocol has been registered in the International Prospective Register of Systematic Reviews (PROSPERO) database, with the registration number CRD420251126527.

### Search strategy

This meta-analysis retrieved relevant randomized controlled trials (RCTs) that explored the efficacy of PD-1/PD-L1 inhibitors combined with multi-targeted anti-angiogenic TKIs in patients with advanced or metastatic NSCLC. The search strategy combined Medical Subject Headings (MeSH) terms and free-text terms as follows: (Immune Checkpoint Inhibitors OR PD-1 inhibitors OR PD-L1 inhibitors OR Sintilimab OR Camrelizumab OR Pembrolizumab OR Nivolumab OR Atezolizumab OR Durvalumab OR Toripalimab OR Penpulimab OR Tislelizumab OR Cindilimab OR Adebrelimab OR Envafolimab) AND (Carcinoma, Non-Small-Cell Lung AND Non-Small Cell Lung Cancer) AND (Angiogenesis Inhibitors OR Anti-Angiogenetic Agents OR Apatinib OR Anlotinib OR Cabozantinib OR Nintedanib OR Lenvatinib). Searches were carried out in the PubMed, EMBASE, Web of Science, ClinicalTrials.gov and the Cochrane library databases in accordance with the established search terms from the establishment of each database to July 2025. And a secondary index of the references of published research was conducted to avoid potential omissions of articles. All studies presented in the article draw on published RCTs. Then, all retrieved records were imported into EndNote 21 for duplicate removal. Two independent reviewers screened the remaining articles based on titles and abstracts. Next, potentially eligible studies underwent full-text review by dual reviewers.

### Selection criteria

Studies included in this meta-analysis were required to meet the following criteria: 1) participants were patients suffered from advanced or metastatic NSCLC; 2) the intervention involved multi-targeted anti-angiogenic TKIs combined with PD-1/PD-L1 inhibitors; 3) the control group included chemotherapy or PD-1/PD-L1 inhibitors with or without chemotherapy; 4) the outcome measures included PFS or OS; 5) the study design was a RCT.

Studies were excluded based on the following criteria: 1) the full text was unavailable; 2) the data from the studies cannot be statistically analyzed.; 3) if multiple publications arose from the same patient cohort, only the one with the most complete data or the most recent publication was selected for inclusion.

### Data extraction and quality assessment

Data extraction was performed independently by the two reviewers using a predefined form, which collected study characteristics (authors, clinical trial number, year of publication, trial name, country, recruitment time, blinding status), participant characteristics (sample size, histology, interventions, line of treatment) and primary outcomes. Any disagreements were resolved through discussion; if consensus could not be reached, a third reviewer was consulted for arbitration. The Cochrane Risk of Bias Assessment Tool was used to conduct risk assessment of RCTs, mainly including the following seven aspects: (1) Selection bias: random sequence generation; (2) Selection bias: allocation hiding; (3) Implementation bias: trial blindness to researchers and subjects; (4) Measurement bias: blind evaluation of study outcomes; (5) Follow-up bias: completeness of outcome data; (6) Reporting bias: selective reporting of study results; (7) Other bias: bias in other ways.

### Statistical analysis

Statistical analysis was conducted using Stata 12.0. The PFS and OS were assessed using hazard ratio (HR) and 95% confidence interval (95%CI). An HR<1 indicates that the intervention is associated with improved outcomes, whereas an HR>1 suggests a potential detrimental effect on prognosis. In this meta-analysis, heterogeneity across studies was assessed using Chi-square tests and quantified with I² statistics. An I² value>50% was considered to indicate high heterogeneity; 25%-50%, moderate heterogeneity; and<25%, low heterogeneity. Considering the inherent heterogeneity among studies, such as the differences in tumor histological types in the population and the types and dosages of drugs in intervention measures, this study adopts a random-effects model to enhance the credibility of the results. Prespecified subgroups were age, sex, Eastern Cooperative Oncology Group (ECOG) performance status (PS), smoking history, metastasis status, PD-L1 expression, race, histology and therapeutic status. Publication bias was evaluated using Begg’s test, and outcome robustness was assessed via leave-one-out sensitivity analysis. All analyses employed two-sided statistical tests, and p<0.05 was considered statistically significant.

## Results

### Results of literature search.

**Figure 1:**
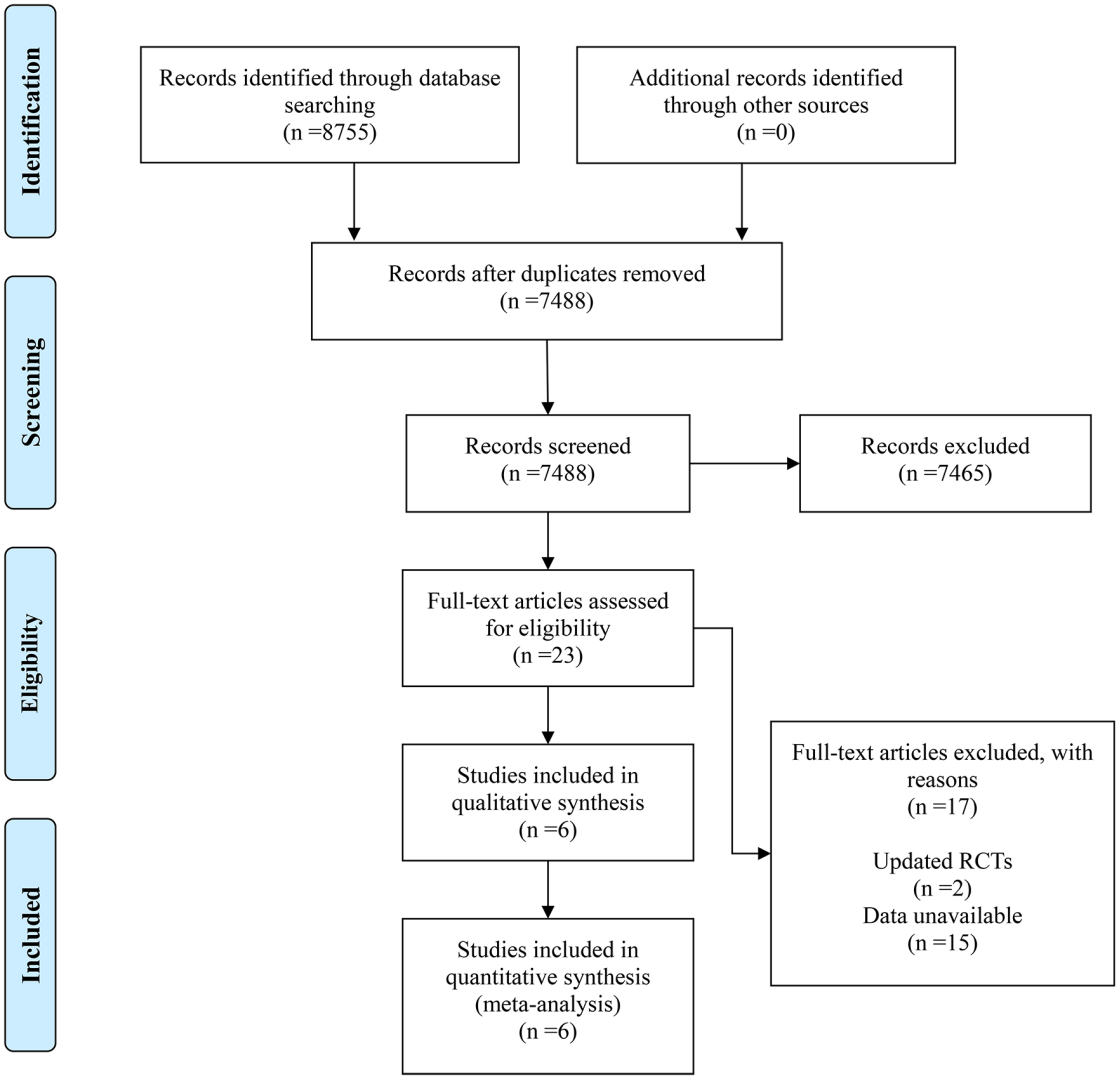
Flow chart representing details of the study selection.

A total of 8,755 articles were retrieved from PubMed, EMBASE, Web of Science, ClinicalTrials.gov, and the Cochrane Library databases, without additional sources consulted. After removing duplicates, 7,488 articles remained. By reviewing titles and abstracts, 7,465 articles were excluded. Full-text review of the remaining 23 articles led to the exclusion of 17 (2 were updated RCTs, and 15 lacked available data). Ultimately, 6 RCTs were included in the meta-analysis (12, 13, 17–20). The entire process is illustrated in **Figure 1**.

### Characteristics of studies and participants

Six RCTs published between 2024 and 2025 included 2,787 participants, with 1,388 assigned to the intervention group and 1,399 to the control group. In these studies, the intervention served as first-line therapy in three trials and as non-first-line therapy in the remaining three. All patients in the intervention group of this study received treatment with a PD-1/PD-L1 inhibitor combined with a multi-targeted anti-angiogenic TKI (targeting VEGFR, FGFR, PDGFR, etc.), with or without chemotherapy. The PD-1/PD-L1 inhibitors used included pembrolizumab (n=3), sintilimab (n=1), atezolizumab (n=1), and nivolumab (n=1); the multi-targeted anti-angiogenic TKIs used included lenvatinib (n=3), anlotinib (n=1), cabozantinib (n=1), and sitravatinib (n=1). The control group treatment regimen consisted of chemotherapy (n=4), monotherapy with a PD-1/PD-L1 inhibitor (n=1), or combination therapy with a PD-1/PD-L1 inhibitor and chemotherapy (n=1). Additionally, four studies employed an “open label” design, while two studies used a “double-blind” design. Study characteristics are summarized in **Table 1**, with additional participant details provided in **Supplementary Table 1**.

**Table 1:** Basic characteristics of randomized controlled trials.

Clinical trial number	Year	Trial name	Intervene group (no. of patients) Control group (no. of patients)	Tx line	Primary outcomes	Blind
NCT03976375	2025	LEAP-008	Lenvatinib + Pembrolizumab (n=185) Docetaxel (n=189)	≥2	OS, PFS	Open-label
NCT03829319	2025	LEAP-006	Lenvatinib + Pembrolizumab + Pemetrexed + Carboplatin/Cisplatin (n=375) Placebo + Pembrolizumab + Pemetrexed + Carboplatin/Cisplatin (n=373)	1	OS, PFS	Double-blind
NCT04124731	2025	SUNRISE	Sintilimab + Anlotinib (n=49) Pemetrexed/Gemcitabine + Carboplatin (n=50)	1	ORR	Open-label
NCT03829332	2024	LEAP-007	Lenvatinib + Pembrolizumab (n=309) Placebo + Pembrolizumab (n=314)	1	OS, PFS	Double-blind
NCT04471428	2024	CONTACT-01	Atezolizumab + Cabozantinib (n=186) Docetaxel (n=180)	≥2	OS	Open-label
NCT03906071	2024	SAPPHIRE	Nivolumab + Sitravatinib (n=284) Docetaxel (n=293)	≥2	OS	Open-label

TX, treatment; OS, overall survival; PFS, progression-free survival; ORR, objective response rate.

### Results of quality assessment

The Cochrane Risk of Bias Assessment Tool was used to evaluate all included studies. The results of the quality assessment are presented in **Supplementary Figures 1**, **2**. Among these, four studies had a high risk of performance bias due to the lack of blinding for participants and researchers. Two studies had a high risk of detection bias due to the lack of blinding for outcome assessment, and one study had an unclear risk of detection bias as it did not report relevant information. Four studies had an unclear risk of selection bias: one did not report the method for generating random sequences, and three did not report the method for allocation concealment. All studies demonstrated a low risk of attrition bias and reporting bias, and the risk of other biases was unclear for all studies.

### Main outcomes-PFS and OS analysis

**Figure 2:**
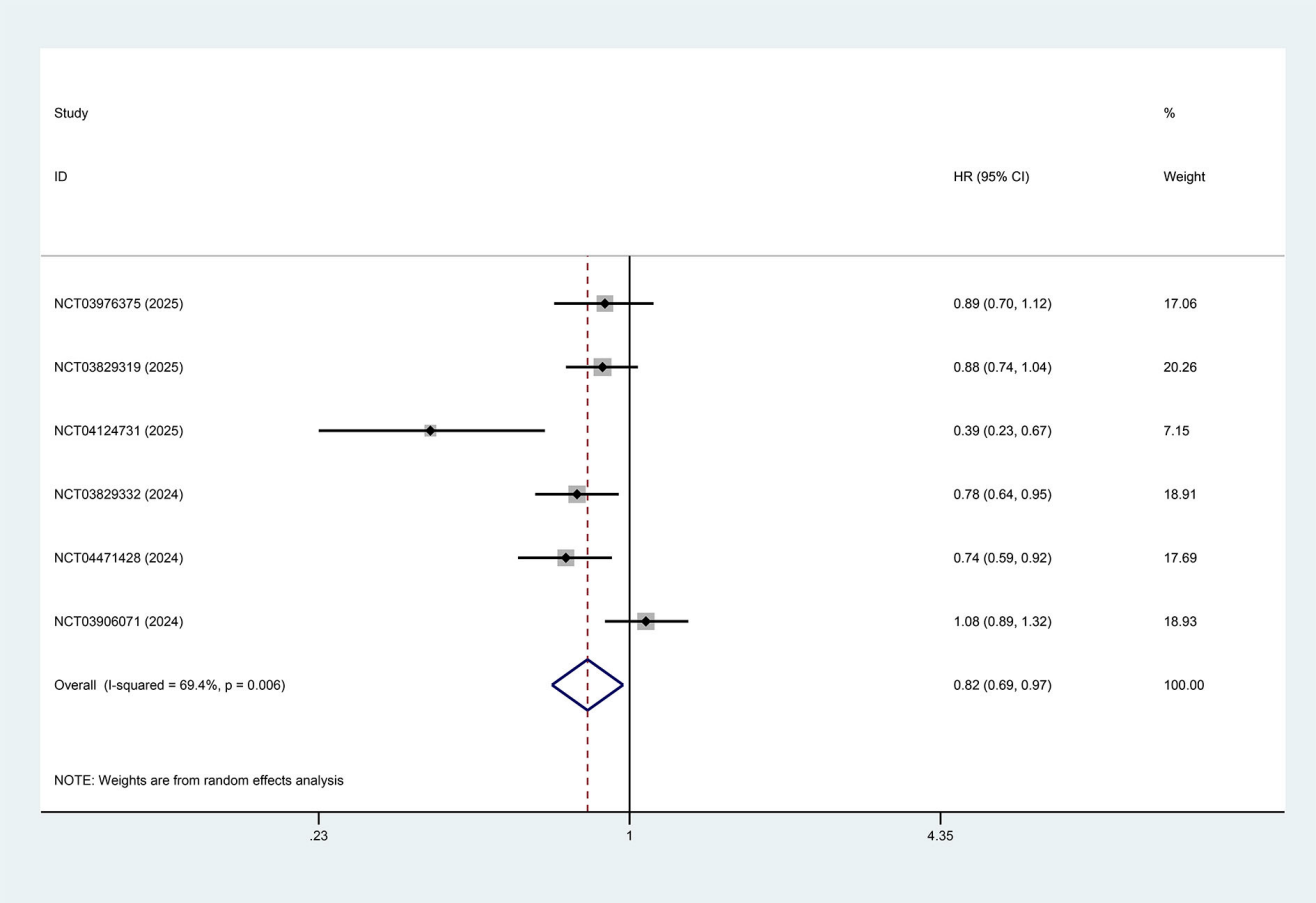
Forest plot showing the effect of PD-1/PD-L1 inhibitors combined with multi-targeted anti-angiogenic TKIs on PFS in patients with advanced or metastatic NSCLC (p=0.021).

**Figure 3:**
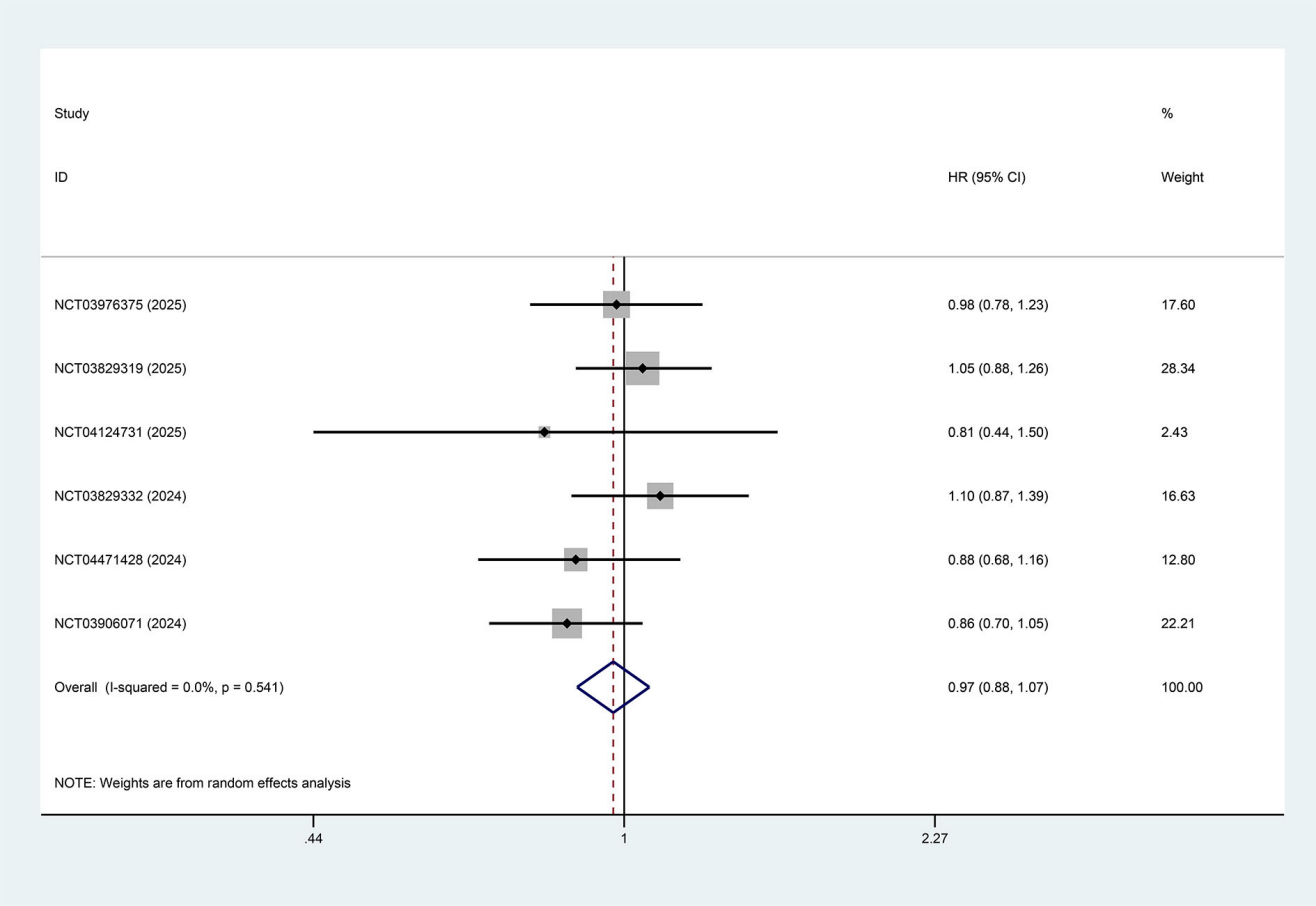
Forest plot showing the effect of PD-1/PD-L1 inhibitors combined with multi-targeted anti-angiogenic TKIs on OS in patients with advanced or metastatic NSCLC (p=0.554).

All studies reported the results of PFS and OS comparing the intervention group (PD-1/PD-L1 inhibitors combined with anti-angiogenic TKIs with or without chemotherapy) with the control group (chemotherapy alone or PD-1/PD-L1 inhibitors with or without chemotherapy). The pooled analysis showed that PFS was significantly improved in the intervention group compared with the control group (HR = 0.82, 95%CI: 0.69-0.97, p=0.021, I²=69.4%), as shown in **Figure 2**. In contrast, no statistically significant difference in OS was observed between the intervention and control groups for patients with advanced or metastatic NSCLC (HR = 0.97, 95%CI: 0.88–1.07, p=0.554, I²=0%), as depicted in **Figure 3**.

*Subgroup analysis-PFS*. Subgroup analysis was conducted as pre-specified to explore sources of heterogeneity. Regarding age, the intervention group demonstrated statistically significant superior PFS compared with the control group in the<65 age cohort (HR = 0.78, 95%CI: 0.64-0.94). However, no statistically significant difference in PFS was observed between the two groups in the≥65 age cohort (HR = 0.85, 95%CI: 0.71-1.02). Regarding ECOG PS, no statistically significant difference in PFS was observed between the two groups among patients with ECOG PS = 0 (HR = 0.84, 95%CI: 0.65-1.08). However, among patients with ECOG PS = 1 (HR = 0.78, 95%CI: 0.64-0.96), the intervention group demonstrated superior PFS compared with the control group.

Regarding metastasis status, no statistically significant difference in PFS was observed between the two groups among patients with liver metastases (HR = 0.79, 95%CI: 0.61-1.04); however, among patients without liver metastases (HR = 0.78, 95%CI: 0.64-0.96), the intervention group demonstrated better PFS than the control group. Similarly, no statistically significant difference in PFS was observed between the two groups among patients with baseline brain metastasis (HR = 0.93, 95%CI: 0.65-1.33). For patients without brain metastases (HR = 0.73, 95%CI: 0.53-1.00, p=0.054), the intervention group showed a numerical but non-significant trend toward improved PFS compared with the control group.

Regarding sex, no statistically significant difference in PFS was observed between the two groups in male patients (HR = 0.78, 95%CI: 0.54-1.12); however, female patients (HR = 0.73, 95%CI: 0.57-0.92) in the intervention group demonstrated superior PFS compared with the control group. Regarding tobacco history, the intervention group demonstrated superior PFS compared with the control group among never-smokers (HR = 0.72, 95%CI: 0.53-0.98); however, among current or former smokers (HR = 0.87, 95%CI: 0.75-1.01), no statistically significant difference in PFS was observed between the two groups. Regarding race, the intervention group demonstrated superior PFS compared with the control group among white patients (HR = 0.85, 95%CI: 0.72-0.99); however, no statistically significant difference in PFS was observed between the intervention and control groups among non-white patients (HR = 1.02, 95%CI: 0.67-1.57).

Regarding PD-L1 expression status, as reflected by tumor proportion score (TPS), no statistically significant difference in PFS was observed between the intervention and control groups in patients with PD-L1<49%. However, in patients with PD-L1≥50%, the intervention group demonstrated superior PFS compared with the control group (PD-L1<1%: HR = 0.83, 95%CI: 0.63-1.07; 1%≤PD-L1<49%: HR = 0.59, 95%CI: 0.34-1.03; PD-L1≥50%: HR = 0.78, 95%CI: 0.63-0.96, respectively).Within the histological subtype subgroup, the intervention group demonstrated superior PFS compared with the control group in patients with non-squamous NSCLC (HR = 0.82, 95%CI: 0.66-1.01, p=0.064), though this difference did not reach statistical significance. In patients with squamous NSCLC (HR = 0.84, 95%CI: 0.59-1.20), no statistically significant difference in PFS was observed between the intervention and control groups.

In the therapeutic status subgroup, patients receiving the intervention as first-line therapy (HR = 0.72, 95%CI: 0.54-0.97) demonstrated superior PFS in the intervention group compared with the control group. However, no statistically significant difference was observed between the intervention and control groups among patients receiving the intervention as second-line or later therapy (HR = 0.90, 95%CI: 0.79-1.03). In the treatment strategy subgroup, no statistically significant difference was observed between the PD-1/PD-L1 inhibitors combined with anti-angiogenic TKIs and chemotherapy among patients (HR = 0.78, 95%CI: 0.58-1.05). Detailed results of all subgroup analyses are presented in **Table 2**.

**Table 2:** Subgroup analysis of progression-free survival.

Subgroup	No. of studies	HR	95%CI	P	Heterogeneity	
					I^2^	P
Age (years)						
	4	0.78	(0.64-0.94)	0.010	26.4%	0.253
≥65	4	0.85	(0.71-1.02)	0.076	22.8%	0.274
ECOG PS						
0	3	0.84	(0.65-1.08)	0.180	39.6%	0.191
1	4	0.78	(0.64-0.96)	0.018	53.7%	0.091
Liver metastases						
Yes	4	0.79	(0.61-1.04)	0.092	0	0.826
No	4	0.78	(0.64-0.96)	0.019	60.3%	0.056
Brain metastases						
Yes	3	0.93	(0.65-1.33)	0.699	0	0.478
No	3	0.73	(0.53-1.00)	0.054	72.1%	0.028
Sex						
Male	3	0.78	(0.54-1.12)	0.180	74.3%	0.020
Female	3	0.73	(0.57-0.92)	0.008	0	0.659
PD-L1 status						
	2	0.83	(0.63-1.07)	0.154	0	0.704
1-49%	3	0.59	(0.34-1.03)	0.063	85.3%	0.001
≥50%	4	0.78	(0.63-0.96)	0.019	0	0.801
Tobacco history						
Never	4	0.72	(0.53-0.98)	0.040	29.0%	0.238
Former or Current	3	0.87	(0.75-1.01)	0.072	0	0.414
Race						
White	2	0.85	(0.72-0.99)	0.042	0	0.528
All others	2	1.02	(0.67-1.57)	0.920	50.2%	0.156
Histology						
Nonsquamous	5	0.82	(0.66-1.01)	0.064	71.9%	0.007
Squamous	3	0.84	(0.59-1.20)	0.342	44.5%	0.165
Treatment line						
1st line	3	0.72	(0.54-0.97)	0.029	75.6%	0.017
≥2nd line	3	0.90	(0.72-1.12)	0.338	68.1%	0.044
Treatment strategy						
ICI + TKI vs CT	4	0.78	(0.58-1.05)	0.107	80.5%	0.002

ECOG PS, Eastern Cooperative Oncology Group performance status; PD-L1, programmed death ligand 1; ICI, immune checkpoint inhibitor (PD-1/PD-L1 inhibitor); TKI, tyrosine kinase inhibitor (anti-angiogenic tyrosine kinase inhibitor); CT, chemotherapy; HR, hazard ratio; CI, confidence interval.

*Subgroup analysis-OS*. Subgroup analyses were performed for OS based on pre-specified factors, including age, presence of liver metastases, presence of brain metastases, sex, PD-L1 expression status, tobacco history, race, histology, therapeutic status and treatment strategy. The results consistently demonstrated no statistically significant OS benefit of the intervention group (PD-1/PD-L1 inhibitors plus multi-targeted anti-angiogenic TKIs with or without chemotherapy) over the control group across all these subgroups (all p>0.05). The results of the subgroup analyses are summarized in **Table 3**.

**Table 3:** Subgroup analysis of overall survival.

Subgroup	No. of studies	HR	95%CI	P	Heterogeneity	
					I^2^	P
Age (years)						
	6	0.90	(0.79-1.03)	0.137	0	0.836
≥65	6	1.06	(0.93-1.21)	0.368	0	0.641
ECOG PS						
0	5	1.03	(0.87-1.23)	0.698	0	0.951
1	6	0.97	(0.86-1.09)	0.556	6.2%	0.377
Liver metastases						
Yes	4	0.89	(0.68-1.18)	0.421	0	0.733
No	4	1.05	(0.92-1.19)	0.448	0	0.716
Brain metastases						
Yes	4	1.03	(0.73-1.45)	0.862	29.1%	0.238
No	4	0.95	(0.84-1.08)	0.436	0	0.444
Sex						
Male	5	0.92	(0.74-1.15)	0.464	61.7%	0.034
Female	5	0.95	(0.75-1.21)	0.686	32.6%	0.204
PD-L1 status						
	3	0.95	(0.76-1.20)	0.682	0	0.897
1-49%	4	0.98	(0.79-1.23)	0.892	17.3%	0.304
≥50%	5	1.07	(0.86-1.33)	0.537	0	0.648
Tobacco history						
Never	6	0.94	(0.74-1.21)	0.651	0	0.439
Former or Current	3	1.05	(0.91-1.22)	0.495	0	0.706
Race						
White	3	0.95	(0.82-1.10)	0.474	0	0.790
All others	2	1.20	(0.90-1.60)	0.213	0	0.711
Histology						
Nonsquamous	6	0.97	(0.87-1.07)	0.506	0	0.832
Squamous	4	0.97	(0.71-1.33)	0.850	39.6%	0.174
Treatment line						
1st line	3	1.05	(0.92-1.21)	0.464	0	0.658
≥2nd line	3	0.90	(0.79-1.03)	0.131	0	0.686
Treatment strategy						
ICI + TKI vs CT	4	0.90	(0.79-1.02)	0.106	0	0.832

ECOG PS, Eastern Cooperative Oncology Group performance status; PD-L1, programmed death ligand 1; ICI, immune checkpoint inhibitor (PD-1/PD-L1 inhibitor); TKI, tyrosine kinase inhibitor (anti-angiogenic tyrosine kinase inhibitor); CT, chemotherapy; HR, hazard ratio; CI, confidence interval.

### Sensitivity analysis and publication bias

To assess potential publication bias in PFS outcomes, the Begg’s test was applied. Analysis revealed that the funnel plot exhibited broadly symmetric characteristics, indicating no significant publication bias for this endpoint (p=0.133). Detailed results are presented in **Supplementary Figure 3**. To validate the robustness of the PFS analysis, sensitivity analyses were conducted by sequentially excluding individual studies, demonstrating good stability, as shown in **Supplementary Figure 4**. For potential publication bias in OS outcomes, Begg’s test was similarly applied. The assessment revealed that the funnel plot for OS showed a broadly symmetric distribution, indicating no significant publication bias (p>0.999). See **Supplementary Figure 5** for details. Subsequent sensitivity analyses excluding individual studies sequentially were used to confirm the robustness of OS results, demonstrating good stability, as shown in **Supplementary Figure 6**.

## Discussion

This meta-analysis identified a phenomenon: in patients with advanced or metastatic NSCLC, the combination of PD-1/PD-L1 inhibitors with multi-targeted anti-angiogenic TKIs significantly improved PFS. Notably, this PFS improvement was primarily confined to a specific patient profile: younger, female, non-smoking white individuals; those with non-squamous NSCLC, high PD-L1 expression (TPS≥50%), an ECOG PS of 1, and no liver or brain metastases; and those receiving the combination as first-line therapy. However, this advantage did not translate into a significant OS benefit.

The PFS benefit observed in our study is biologically plausible and supports the synergistic association between anti-angiogenic TKIs and PD-1/PD-L1 inhibitors. PD-1/PD-L1 inhibitors exert antitumor effects by blocking PD-1/PD-L1 signaling and thereby reactivating antitumor T cell responses (21). However, the efficacy of PD-1/PD-L1 inhibitors depends not only on the reactivation of antitumor T cell responses, but also on the ability of effector T cells to infiltrate the TME and remain functionally active within it. In this context, multi-targeted anti-angiogenic TKIs may provide an important complementary role. By simultaneously targeting multiple angiogenesis-related pathways, including vascular endothelial growth factor (VEGF)/VEGFR, fibroblast growth factor (FGF)/FGFR, and platelet-derived growth factor (PDGF)/PDGFR (7), these agents may not only limit compensatory pro-angiogenic signaling (22, 23) but also enhance the activity of PD-1/PD-L1 inhibitors by facilitating T-cell infiltration and alleviating T-cell dysfunction.

First, anti-angiogenic TKIs may help overcome barriers that prevent T cells from trafficking into the TME. Aberrant tumor angiogenesis generates a hostile TME characterized by hypoxia, acidosis, and elevated interstitial fluid pressure, which not only restrict the penetration of anticancer agents and immune cells into tumor tissue, but also compromise their antitumor activity within the TME (24, 25). In addition, aberrant tumor vasculature limits immune cell infiltration through both structural and functional mechanisms: the disorganized vascular network hinders T cell entry, while VEGF downregulates endothelial adhesion molecules such as intercellular adhesion molecule 1 (ICAM1) and vascular cell adhesion molecule 1 (VCAM1), thereby impairing transendothelial T cell trafficking (26, 27). In parallel, stromal remodeling provides an additional obstacle to T cell infiltration. Signaling through pathways such as FGF/FGFR and PDGF/PDGFR can promote fibroblast recruitment and expansion, as well as their transition into cancer-associated fibroblasts (CAFs), which generate dense extracellular matrix barriers that physically hinder T cell penetration into the tumor (28, 29). Activated CAFs also secrete C-X-C motif ligand 12 (CXCL12), which can further promote T cell exclusion from tumor tissue (28, 30). In addition, PDGF/PDGFR signaling contributes to extracellular matrix remodeling and elevated interstitial fluid pressure, whereas inhibition of PDGFR has been shown to reduce interstitial pressure and improve intratumoral drug delivery (29). Together, these findings suggest that anti-angiogenic TKIs may create a more accessible TME for T cell entry.

Second, anti-angiogenic TKIs may also enhance the efficacy of PD-1/PD-L1 inhibitors by attenuating T cell dysfunction and reversing immune suppression. VEGF not only promotes aberrant angiogenesis, but also exerts immunosuppressive effects by recruiting regulatory T cells (Tregs) (31), myeloid-derived suppressor cells (MDSCs) (32), and M2-like tumor-associated macrophage (TAM) (33) into the TME. In addition, VEGF impairs dendritic cell (DC) maturation, antigen presentation, and the differentiation of CD4^+^ and CD8^+^ T cells (24, 34), thereby weakening effective antitumor immune priming. Consistent with this concept, preclinical studies suggest that FGFR inhibition may partially reverse immune suppression within the TME by increasing effector T cell infiltration, reducing suppressive immune-cell subsets, and enhancing the breadth of antitumor T cell responses (28). When combined with PD-1 inhibitors, these effects may further strengthen immune-mediated tumor control.

Taken together, anti-angiogenic TKIs may enhance the efficacy of PD-1/PD-L1 inhibitors by remodeling the suppressive TME, overcoming barriers to T-cell infiltration, and attenuating T-cell dysfunction, which may partly explain the PFS benefit observed in our study.

Immunotherapy has revolutionized NSCLC treatment strategies, and PD-L1 expression in tumor tissue determined by immunohistochemistry is a critical biomarker for predicting therapeutic response (35). For patients with advanced or metastatic NSCLC without driver gene mutations, PD-1/PD-L1 inhibitors are a preferred treatment option, and their efficacy is associated with tumor PD-L1 expression level. In patients with high PD-L1 expression (TPS≥50%), PD-1/PD-L1 inhibitor monotherapy is the preferred first-line treatment (36, 37). Our meta-analysis further showed that these PD-L1-high patients also derived a PFS benefit from the addition of anti-angiogenic TKIs, whereas no significant benefit was observed in patients with PD-L1 TPS<50%.

High PD-L1 expression often indicates an inflammatory TME, characterized by tumor-infiltrating lymphocytes (TILs), high-density IFN-γ-producing CD8^+^ T cells, and PD-L1-expressing tumor cells (38). This suggests intense immune-tumor conflict, frequently manifested as crosstalk between the immune microenvironment and angiogenesis (39). In such cases, incorporating anti-angiogenic TKIs into PD-1/PD-L1 inhibitors can simultaneously promote tumor vascular normalization to enhance immune cell infiltration while suppressing immunosuppressive cells and activating immune cells, thereby amplifying immunotherapy efficacy (39). Conversely, in tumors with relatively low PD-L1 expression (TPS<50%), the immune microenvironment remains cold, lacking intense immune-tumor conflict. Even if anti-angiogenic TKIs improve the immunosuppressive microenvironment and promote vascular normalization, the efficacy of PD-1/PD-L1 inhibitors may still remain limited because immune cell infiltration and activation are insufficient. A clinical study partially elucidated this mechanism: in cold tumors with poor response, a robust structural resistance network exists, centered on TAMs and maintained through VEGF signaling compensation and intercellular communication (40). This network renders anti-angiogenic TKIs ineffective at creating an environment conducive to T cell activity and proliferation (40). Ultimately, the efficacy of PD-1/PD-L1 inhibitors combined with anti-angiogenic TKIs did not show further improvement. Thus, the findings of this meta-analysis further reinforce the central role of PD-L1 expression levels as a biomarker for precision screening of populations benefiting from a combination of PD-1/PD-L1 inhibitors and anti-angiogenic TKIs. This suggests that for patients with advanced or metastatic NSCLC exhibiting high PD-L1 expression (TPS≥50%), adding anti-angiogenic TKIs to PD-1/PD-L1 inhibitor monotherapy may be considered.

Previous studies have shown that PD-1/PD-L1 inhibitors often benefits male smokers with squamous NSCLC, likely due to high PD-L1 expression. However, in this study, the beneficiary population was precisely the opposite—female, non-smoking, non-squamous patients demonstrated benefit. This may be related to the following possible explanations. Recent epidemiological data indicate a rapid global increase in the proportion of young, non-smoking female lung adenocarcinoma patients among NSCLC cases (41–43). This cohort is characterized by high-frequency epidermal growth factor receptor (EGFR) mutations, which are typically associated with limited immunotherapy response (44, 45). Notably, a study of East Asian non-smoking lung adenocarcinoma patients revealed an additional significant source of genetic mutations in young, non-smoking, female lung adenocarcinoma patients, closely linked to apolipoprotein B mRNA editing enzyme catalytic polypeptide like (APOBEC) (46). By the way, APOBEC-related mutational processes may show partial mutual exclusivity with EGFR mutations. Therefore, mutations in this population of East Asian, young, non-smoking, female lung adenocarcinoma patients can primarily include—EGFR-mutant subtype and APOBEC-mutant subtype (46). The APOBEC-mutant subtype has been reported to exhibit higher neoantigen mutation rates, leading to immune activation and infiltration, and theoretically may be more likely to benefit from PD-1/PD-L1 inhibitor immunotherapy (46, 47). One study demonstrated that in APOBEC-type patients, tumor genes are enriched in immune-related pathways, with abundant CD8^+^ T cells and memory CD4^+^ T cells present (48). Tumors tend to display a relatively inflamed immune phenotype, making them theoretically more likely to benefit from PD-1/PD-L1 inhibitor therapy. However, APOBECs also represent a double-edged sword. The continuous subclonal driver mutations generated by APOBECs directly seed acquired resistance (49) and intratumoral heterogeneity (50), rendering tumors more complex and difficult to treat. Anti-angiogenic TKIs, by multi-targeted blockade of angiogenesis pathways, represent a potential pan-cancer therapeutic approach (7, 51). Consistent with the above mechanistic rationale, anti-angiogenic TKIs may act synergistically with PD-1/PD-L1 inhibitors. Theoretically, the combination of PD-1/PD-L1 inhibitors with anti-angiogenic TKIs may be of particular interest in a subset of female, non-smoking, non-squamous patients who may harbor APOBEC-associated molecular features. However, this possibility should be regarded as hypothesis-generating rather than evidence-confirmed, because the included RCTs did not provide direct baseline molecular data on APOBEC-associated mutational status. In addition, although the non-squamous subgroup in our analysis showed a borderline result (p=0.064), this finding may still indicate a possible trend toward benefit in non-squamous patients. At the same time, the limited sample size and heterogeneity (I^2^ = 71.9%) across studies may have contributed to the borderline result. Therefore, our findings are insufficient to conclude that non-squamous NSCLC represents a clearly defined beneficiary subgroup, and they do not directly demonstrate benefit in an APOBEC-associated population. Instead, these results provide a rationale for future molecularly stratified studies to determine whether a subset of female, non-smoking, non-squamous patients, including those with APOBEC-associated features, may derive particular benefit from PD-1/PD-L1 inhibitors plus anti-angiogenic TKIs.

Our subgroup analysis also revealed significant differences in treatment benefits across racial groups. Among patients with EGFR wild-type NSCLC, white patients appeared to derive greater benefit from the combination therapy of PD-1/PD-L1 inhibitors plus anti-angiogenic TKIs compared with non-white patients. Given potential unmeasured confounders and the exploratory nature of subgroup analyses, this observation should be interpreted with caution. The underlying biological or epidemiological determinants for this disparity remain to be elucidated.

Our subgroup analysis revealed that patients with better performance status (ECOG PS = 0) appeared to derive less benefit from the combination regimen than those with slightly poorer status (ECOG PS = 1). This finding contradicts the conventional expectation that better condition correlates with better response (52). We propose this may stem from the need for particularly careful risk-benefit balancing in treatment decisions for advanced, driver mutation-negative NSCLC patients with poor performance status. Even regimens demonstrating superior efficacy may carry toxicity levels intolerable for this cohort, including those with ECOG PS = 1. Studies indicate that for ECOG PS = 1 patients, regimens incorporating chemotherapy not only fail to deliver efficacy but also impose toxicity, compromising PFS (53). This presents an opportunity for PD-1/PD-L1 inhibitors combined with anti-angiogenic TKIs. For ECOG PS = 0 patients, their better performance status allows them to tolerate more intensive regimens, such as PD-1/PD-L1 inhibitors combined with chemotherapy (53). However, caution is warranted in interpreting these findings. This outcome likely stems from the fact that the majority of patients enrolled in the clinical trials were PS = 1, resulting in limited and unstable data for PS = 0 patients. For instance, PS = 1 patients constituted the overwhelming majority (98%) in the SUNRISE study (18). Therefore, current analyses more accurately reflect the efficacy of this therapy in PS = 1 patients. However, this does not negate the potential efficacy of PD-1/PD-L1 inhibitor plus anti-angiogenic TKI therapy in PS = 0 patients.

The brain is a well-recognized immune-privileged organ, and the unique biological features of brain metastases may partly explain the differential benefit observed in this subgroup analysis. Owing to the presence of the blood-brain barrier (BBB), both immune cells and many therapeutic agents have limited access to intracranial lesions (54). Even when the BBB is disrupted by tumor progression, brain metastases may still establish a highly specialized immunosuppressive microenvironment by co-opting infiltrating immune cells and promoting local immune tolerance. In addition, brain metastatic lesions generally contain fewer T cells than primary tumors (55), suggesting that the baseline immune context within the brain may be less favorable for PD-1/PD-L1 inhibitors. In this setting, although anti-angiogenic TKIs may partially remodel the TME by improving vascular abnormalities and alleviating local immune suppression, such effects may be insufficient to fully overcome the restricted immune-cell access and profound local immune exclusion associated with brain metastases. By contrast, in patients without brain metastases, the absence of these intracranial barriers may allow the microenvironment-remodeling effects of anti-angiogenic TKIs to more effectively support PD-1/PD-L1 inhibitors, thereby translating into greater clinical benefit. In our meta-analysis, patients without brain metastases appeared to derive greater benefit from the combination of PD-1/PD-L1 inhibitors and anti-angiogenic TKIs than those with brain involvement. Although this subgroup difference did not reach conventional statistical significance, the borderline P value (p=0.054) may still suggest a potentially meaningful clinical trend. One possible explanation is that the current analysis was underpowered because of limited sample size and substantial heterogeneity (I^2^ = 72.1%) among the included studies. Therefore, this finding should be interpreted cautiously rather than as definitive evidence of subgroup-specific benefit. Nevertheless, it may still provide clinically relevant insight by suggesting that the efficacy of this combination strategy could depend, at least in part, on whether the metastatic site permits sufficient immune-cell access and effective tumor microenvironment remodeling. This issue warrants further validation in larger, prospectively stratified studies.

Although liver metastases occur infrequently in NSCLC, their presence often portends extremely poor prognosis, making immunotherapy a critical treatment option (56). Nevertheless, liver metastases remain an independent predictor of poor immunotherapy response compared with the overall population (57). This phenomenon may relate to the liver’s inherent immune tolerance and its systemic suppression of both local and systemic immune microenvironments (58, 59). One study demonstrated that liver metastases siphon activated CD8^+^ T cells from systemic circulation, where they undergo apoptosis after interacting with FasL^+^CD11b^+^F4/80^+^ monocyte-derived macrophages in the liver, resulting in both local and systemic immune suppression (60). Although anti-angiogenic TKIs can normalize tumor vasculature to promote cytotoxic T cell infiltration and partially reverse the immunosuppressive microenvironment, systemic T cell depletion caused by liver metastasis prevents substantial T cell infiltration within the tumor microenvironment. Consequently, the combination of PD-1/PD-L1 inhibitors with anti-angiogenic TKIs fails to demonstrate significant efficacy in NSCLC patients with liver metastasis. Our meta-analysis results support this observation. Furthermore, the control groups in our included studies predominantly received chemotherapy, which can improve the immunosuppressive microenvironment by killing tumor cells and releasing associated antigens, thereby achieving better efficacy (59). In contrast, the combination of PD-1/PD-L1 inhibitors with anti-angiogenic TKIs yields comparatively less pronounced efficacy in NSCLC patients with liver metastases.

The treatment-strategy subgroup analysis should be interpreted with caution, because the regimens used in the control groups varied across the included RCTs. Specifically, the control arms included chemotherapy alone, PD-1/PD-L1 inhibitor monotherapy, and PD-1/PD-L1 inhibitor plus chemotherapy, representing substantially different clinical settings. This heterogeneity was further compounded by differences in treatment line. Among the included studies, four used chemotherapy alone as the control arm, whereas two used PD-1/PD-L1 inhibitor-based regimens; among the chemotherapy-control studies, three were conducted in the second-line or later-line setting and one in the first-line setting, whereas both studies with PD-1/PD-L1 inhibitor-based control arms were first-line trials.

A more finely stratified analysis according to specific control-arm regimens would have been clinically informative. However, some control-arm categories-particularly those involving PD-1/PD-L1 inhibitor-based regimens-contained too few included RCTs to permit a statistically robust formal subgroup analysis, and could therefore only be interpreted descriptively. Among the two first-line studies with PD-1/PD-L1 inhibitor-based control arms, different patterns were observed: in LEAP-007 (12), where the control arm was PD-1/PD-L1 inhibitor monotherapy, a PFS benefit was observed (HR = 0.78; 95%CI: 0.64-0.95); by contrast, in LEAP-006 (19), where the control arm was PD-1/PD-L1 inhibitor plus chemotherapy, no clear PFS benefit was demonstrated (HR = 0.88; 95%CI: 0.74-1.04). This suggests that the incremental contribution of anti-angiogenic TKIs may vary according to the therapeutic backbone of the comparator regimen, although the very limited number of studies precludes firm conclusions.

Within this context, our meta-analysis suggests that PD-1/PD-L1 inhibitors combined with anti-angiogenic TKIs may represent a potentially useful option in selected first-line settings for patients with advanced or metastatic NSCLC lacking actionable driver mutations. This may be related to the ability of anti-angiogenic TKIs to promote vascular normalization, enhance T-cell infiltration, reverse VEGF-mediated immunosuppression, and remodel the tumor immune microenvironment, thereby potentiating PD-1/PD-L1 inhibitors. By contrast, in the second-line or later-line setting after progression on prior PD-1/PD-L1 inhibitor therapy, this strategy appeared less effective, as our subgroup analysis did not show significantly superior survival benefit compared with chemotherapy. This suggests that the proposed synergistic mechanism may be insufficient to overcome the heterogeneous resistance patterns established after prior immunotherapy (61, 62).

A similarly cautious interpretation is required for the comparison against chemotherapy alone. In the treatment-strategy subgroup analysis, PD-1/PD-L1 inhibitors combined with anti-angiogenic TKIs did not show a statistically significant difference compared with chemotherapy (HR = 0.78, 95%CI: 0.58-1.05), although the point estimate suggested a possible trend toward benefit. Because this comparison also included both first-line and second-line studies, its interpretation remains limited by additional clinical heterogeneity. Nevertheless, the PD-1/PD-L1 inhibitor plus anti-angiogenic TKI regimen still provides a chemotherapy-free alternative with efficacy broadly comparable to chemotherapy in the current analysis, and may therefore remain a clinically relevant option for patients who are ineligible for or decline chemotherapy. Future studies should focus on identifying the clinical setting and patient subgroup in which this chemotherapy-free strategy provides the greatest benefit.

This meta-analysis also observed that PFS benefits did not translate into OS benefits. We speculate this may be related to the broad multi-targeted nature of anti-angiogenic TKIs. This characteristic may introduce a series of complex factors that prevent sustained benefits from combination therapy, ultimately limiting significant improvements in OS. First, off-target toxicities play a role. The broad spectrum of treatment-related adverse events (TRAEs) associated with anti-angiogenic TKIs is intrinsically linked to their multi-targeted effects, as more targets often imply greater potential toxicity (63). Higher TRAE incidence rates lead to treatment discontinuation and may impact subsequent therapies (12), thereby compromising OS. In the six studies (12, 13, 17–20) included in our meta-analysis, the treatment discontinuation rate due to TRAEs in the intervention group (PD-1/PD-L1 inhibitors plus anti-angiogenic TKIs) ranged from 17.3% to 37.3%, higher than the 11.2% to 27.7% observed in the control group. and the mortality rate due to TRAEs was 0.4%–5.6%, higher than the 0.6%–2.7% observed in the control group. Furthermore, overuse or prolonged administration of anti-angiogenic TKIs can elevate tumor hypoxia (64), which may induce resistance to PD-1/PD-L1 inhibitors and promote tumor invasion and metastasis (65). Researchers further suggest that the combination of anti-angiogenic drugs and PD-1/PD-L1 inhibitors primarily demonstrates additive rather than synergistic effects (66). In other words, it enhances initial response rates and delays disease progression (improving objective response rate and PFS), but its impact on translating into long-term survival benefits remains limited (no improvement in OS) (66). Beyond pharmacologic mechanisms, factors in clinical practice and study design also significantly contribute to the lack of significant OS outcomes. For instance, in the LEAP-007 trial (12), 14.2% of patients in the lenvatinib + pembrolizumab group received subsequent treatments, compared with 28.0% in the placebo + pembrolizumab control group. In the SUNRISE trial (18), patients in the control group receiving chemotherapy were permitted to cross over to sintilimab monotherapy as part of the trial design. Such differences in subsequent treatment may narrow or even reverse the OS benefit observed in the intervention group compared with the control group. Furthermore, insufficient follow-up duration and the potential carryover effect of immunotherapy may also impact the analysis of OS data. Among the six studies included in our meta-analysis, only CONTACT-01 (13) reported a minimum follow-up of 10.9 months, while the median follow-up in the remaining studies ranged from 15.9 to 36.8 months, with insufficient OS events in some studies. This immature OS data may also influence the OS outcome.

The primary strength of our study lies in conducting a comprehensive and systematic meta-analysis of the efficacy of the specific strategy combining PD-1/PD-L1 inhibitors with anti-angiogenic TKIs in advanced or metastatic NSCLC. While previous meta-analyses (14–16) have reported on PD-1/PD-L1 inhibitor combinations with anti-angiogenic agents, the concept of anti-angiogenic TKIs has often been treated ambiguously. There has been a failure to clearly distinguish between large-molecule monoclonal antibodies with distinct mechanisms of action and clinical characteristics (e.g., bevacizumab) and small-molecule multi-target TKIs (e.g., anlotinib, lenvatinib).

However, this study has several limitations, and the findings should therefore be interpreted with caution. First, the number of included RCTs was limited, and some subgroup analyses were based on only a small number of studies or patients, which may have limited the robustness of these findings. This is particularly relevant for subgroup findings with borderline statistical significance, such as those for patients without brain metastases and for non-squamous NSCLC. For the same reason, although further stratification according to specific control-arm regimens would have been clinically informative, some categories—particularly those using PD-1/PD-L1 inhibitor monotherapy or PD-1/PD-L1 inhibitor plus chemotherapy as the control arm—contained too few studies to permit a statistically robust formal subgroup analysis, and could therefore only be interpreted descriptively. Second, considerable clinical heterogeneity remained across the included trials, including differences in baseline patient characteristics, treatment line, combination regimens, and control-arm design, which complicates interpretation despite the use of a random-effects model and subgroup analyses. Third, some mechanistic interpretations, particularly the inference regarding a possible APOBEC-associated beneficiary subgroup, are hypothesis-generating rather than directly supported by baseline molecular data from the included RCTs, and therefore require dedicated prospective validation. Finally, OS data were still immature in several included studies, and longer follow-up is needed to determine whether the observed PFS benefit will translate into an OS benefit.

## Conclusion

This meta-analysis demonstrates that combining PD-1/PD-L1 inhibitors with multi-targeted anti-angiogenic TKIs significantly improves PFS, but not OS, in patients with advanced or metastatic NSCLC. Exploratory subgroup analyses suggested that the PFS benefit was particularly pronounced in younger patients, females, non-smokers, and those of white race, specifically among individuals with high PD-L1 expression (TPS≥50%), ECOG PS = 1, and without liver or brain metastases. Collectively, these findings position the PD-1/PD-L1 inhibitors combined with multi-targeted anti-angiogenic TKIs as a potential first-line option for driver-negative NSCLC and offer a chemotherapy-free alternative for patients who are ineligible for or decline chemotherapy. These findings should be interpreted as exploratory and require validation in future large, multicenter RCTs.

## Data Availability

The datasets presented in this study can be found in online repositories. The names of the repository/repositories and accession number(s) can be found in the article/**Supplementary Material**.

## Supplementary material

Supplementary Table 1 Characteristics of the randomized controlled trials included in this meta-analysis.

Supplementary Figure 1 Risk of bias assessment in included studies.

Supplementary Figure 2 Summary of risk of bias across studies.

Supplementary Figure 3 Begg’s funnel plot for PFS in the meta-analysis of PD-1/PD-L1 inhibitors plus multi-targeted anti-angiogenic TKIs for advanced or metastatic NSCLC (p=0.133).

Supplementary Figure 4 Plot of the sensitivity analysis regarding the PFS for the PD-1/PD-L1 inhibitors plus multi-targeted anti-angiogenic TKIs in advanced or metastatic NSCLC.

Supplementary Figure 5 Begg’s funnel plot for OS in the meta-analysis of PD-1/PD-L1 inhibitors plus multi-targeted anti-angiogenic TKIs for advanced or metastatic NSCLC (p>0.999).

Supplementary Figure 6 Plot of the sensitivity analysis regarding the OS for the PD-1/PD-L1 inhibitors plus multi-targeted anti-angiogenic TKIs in advanced or metastatic NSCLC.

## Glossary

NSCLC: non-small cell lung cancer
ICIs: immune checkpoint inhibitors
PD-1: programmed cell death protein-1
PD-L1: programmed death-ligand 1
TME: tumor microenvironment
CTLA-4: cytotoxic T-lymphocyte-associated protein 4
TKIs: tyrosine kinase inhibitors
VEGFR: vascular endothelial growth factor receptor
FGFR: fibroblast growth factor receptor
PDGFR: platelet-derived growth factor receptor
PFS: progression-free survival
OS: overall survival
PRISMA: Preferred Reporting Items for Systematic Reviews and Meta-Analyses
PROSPERO: International Prospective Register of Systematic Reviews
RCT: randomized controlled trials
MeSH: Medical Subject Headings
HR: hazard ratio
95%CI: 95% confidence interval
ECOG: Eastern Cooperative Oncology Group
PS: performance status
TPS: tumor proportion score
VEGF: vascular endothelial growth factor
PDGF: platelet-derived growth factor
FGF: fibroblast growth factor
ICAM1: intercellular adhesion molecule 1
VCAM1: vascular cell adhesion molecule 1
CAFs: cancer-associated fibroblasts
CXCL12: C-X-C motif ligand 12
Tregs: regulatory T cells
MDSCs: myeloid-derived suppressor cells
TAM: tumor-associated macrophage
DC: dendritic cell
TILs: tumor-infiltrating lymphocytes
IFN-γ: interferon-gamma
EGFR: epidermal growth factor receptor
APOBEC: apolipoprotein B mRNA editing enzyme catalytic polypeptide like
BBB: blood-brain barrier
TRAE: treatment-related adverse event.
