# Supplementary figures and images for: Efficacy of PD-1/PD-L1 inhibitors combined with multi-targeted anti-angiogenic TKIs in advanced or metastatic NSCLC: A meta-analysis based on RCTs
Source: Front Oncol. 2026 Apr 1;16:1799126. doi: 10.3389/fonc.2026.1799126 (PMC13078995; doi:10.3389/fonc.2026.1799126)

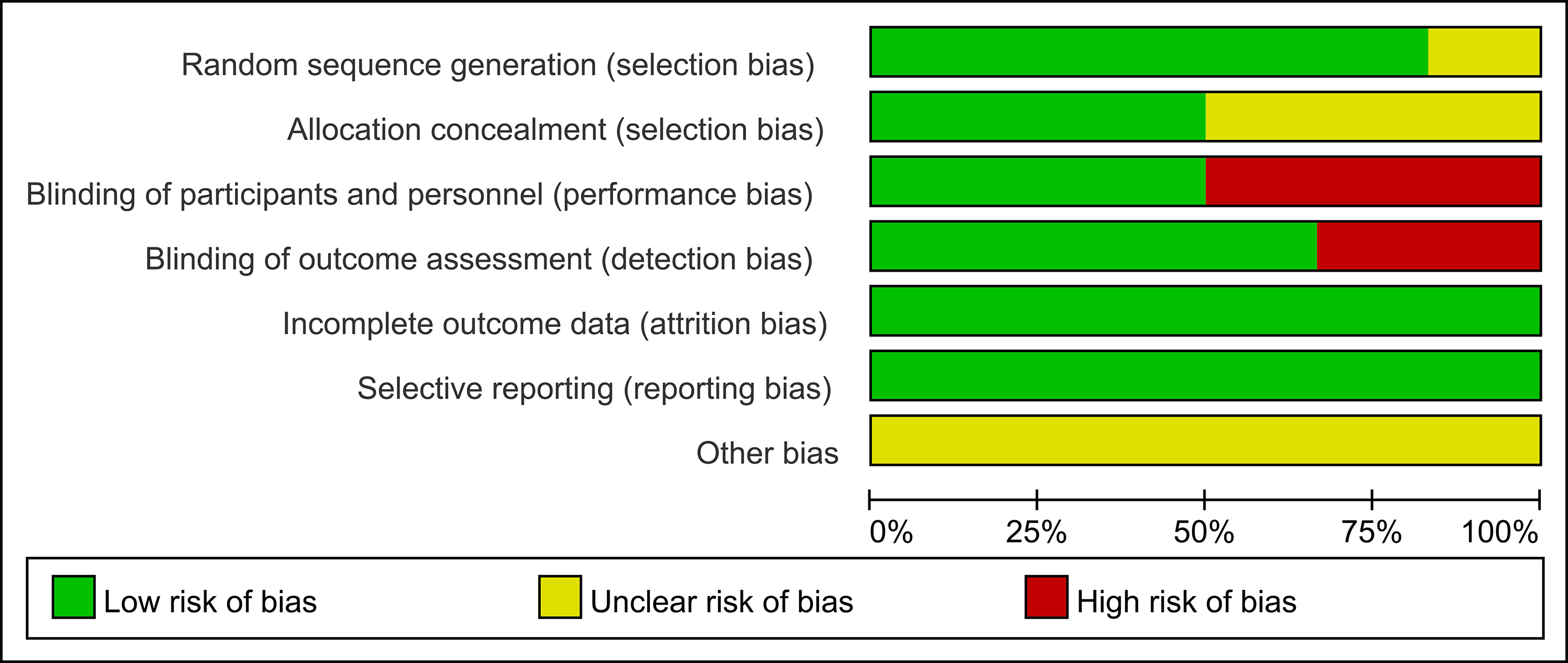

Supplement: Supplementary Table 1 — Characteristics of the randomized controlled trials included in this meta-analysis. [file Image1.tif]

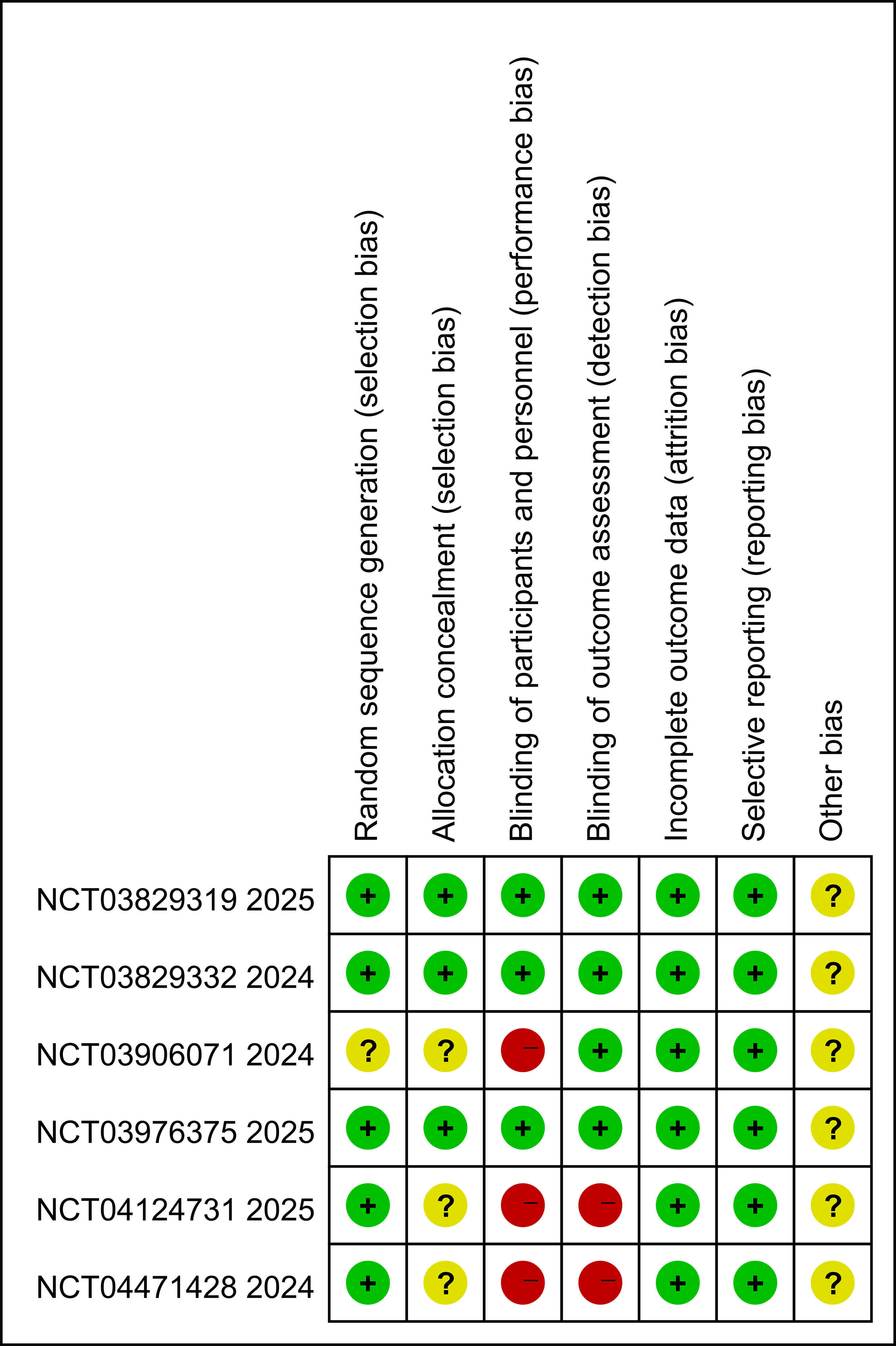

Supplement: Supplementary Figure 2 — Summary of risk of bias across studies. [file Image2.tif]

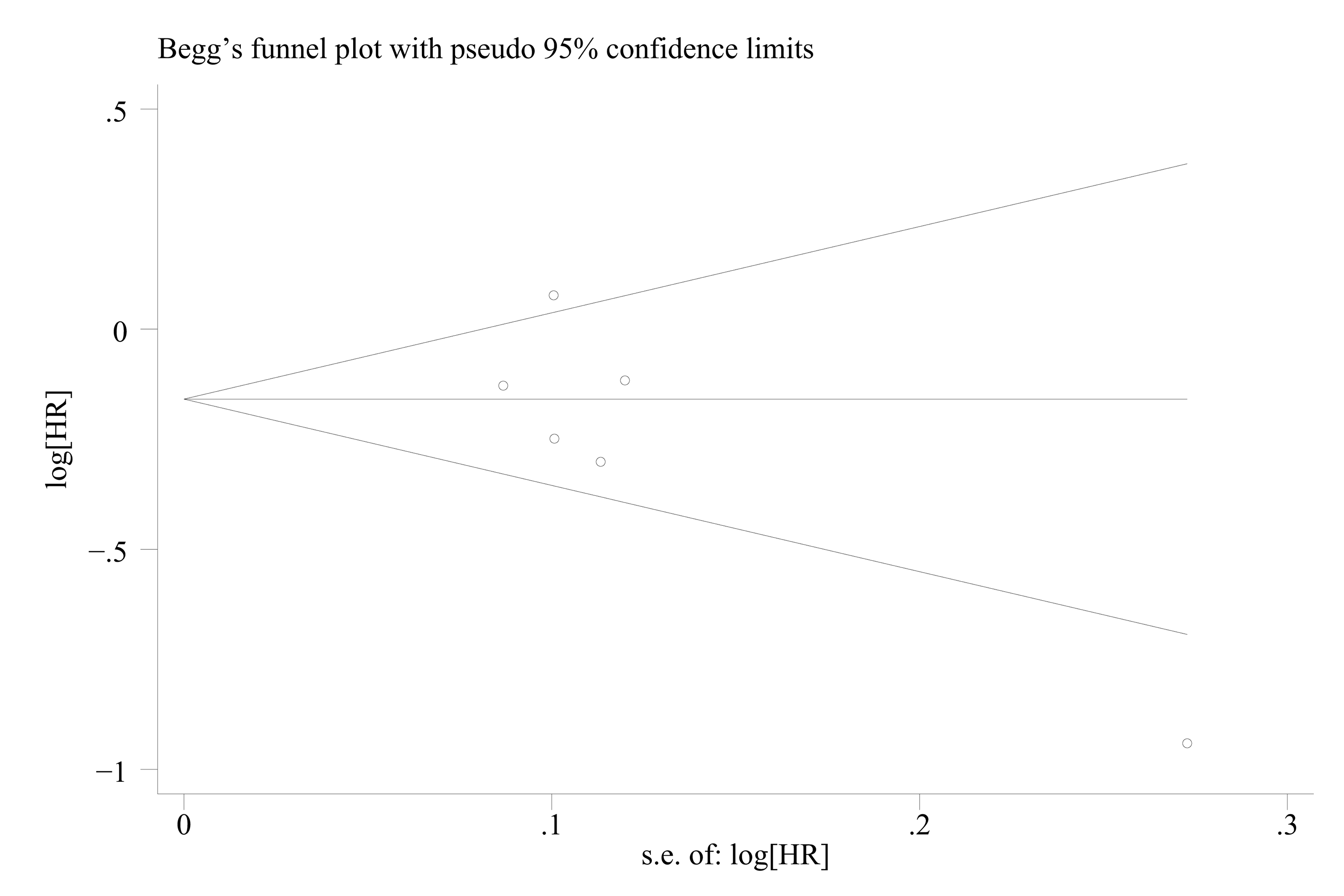

Supplement: Supplementary Figure 3 — Begg’s funnel plot for PFS in the meta-analysis of PD-1/PD-L1 inhibitors plus multi-targeted anti-angiogenic TKIs for advanced or metastatic NSCLC (p=0.133). [file Image3.tif]

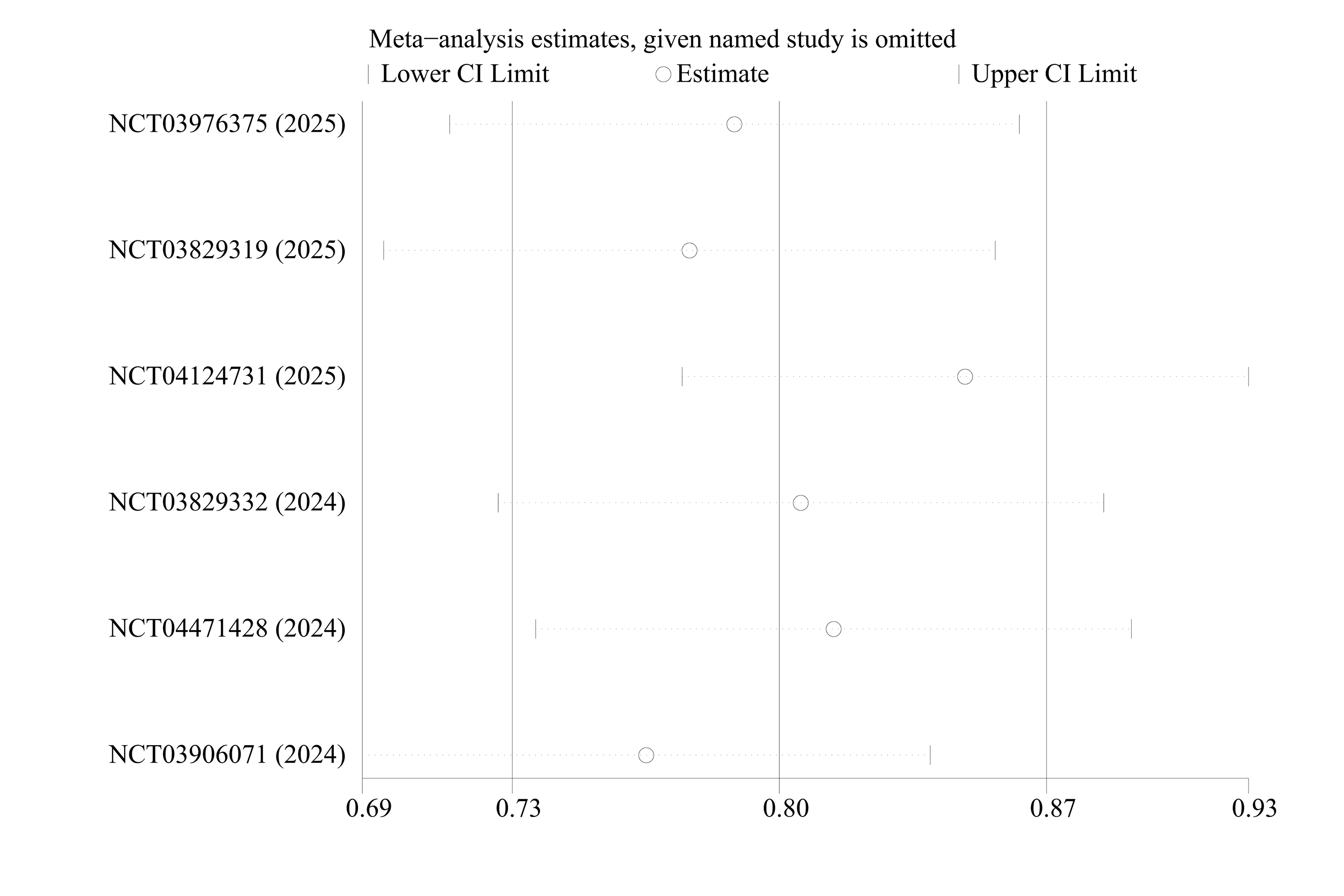

Supplement: Supplementary Figure 4 — Plot of the sensitivity analysis regarding the PFS for the PD-1/PD-L1 inhibitors plus multi-targeted anti-angiogenic TKIs in advanced or metastatic NSCLC. [file Image4.tif]

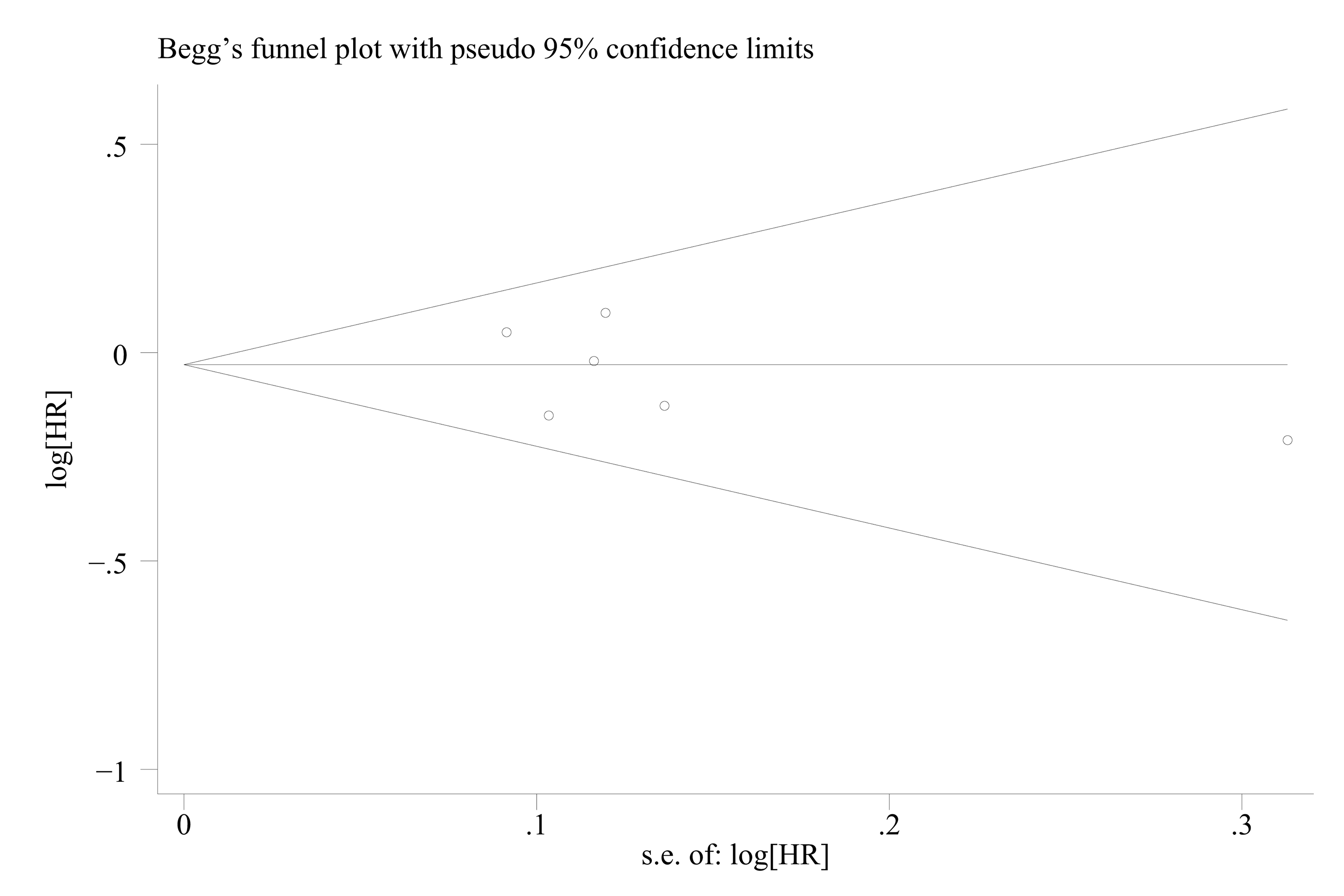

Supplement: Supplementary Figure 5 — Begg’s funnel plot for OS in the meta-analysis of PD-1/PD-L1 inhibitors plus multi-targeted anti-angiogenic TKIs for advanced or metastatic NSCLC (p>0.999). [file Image5.tif]

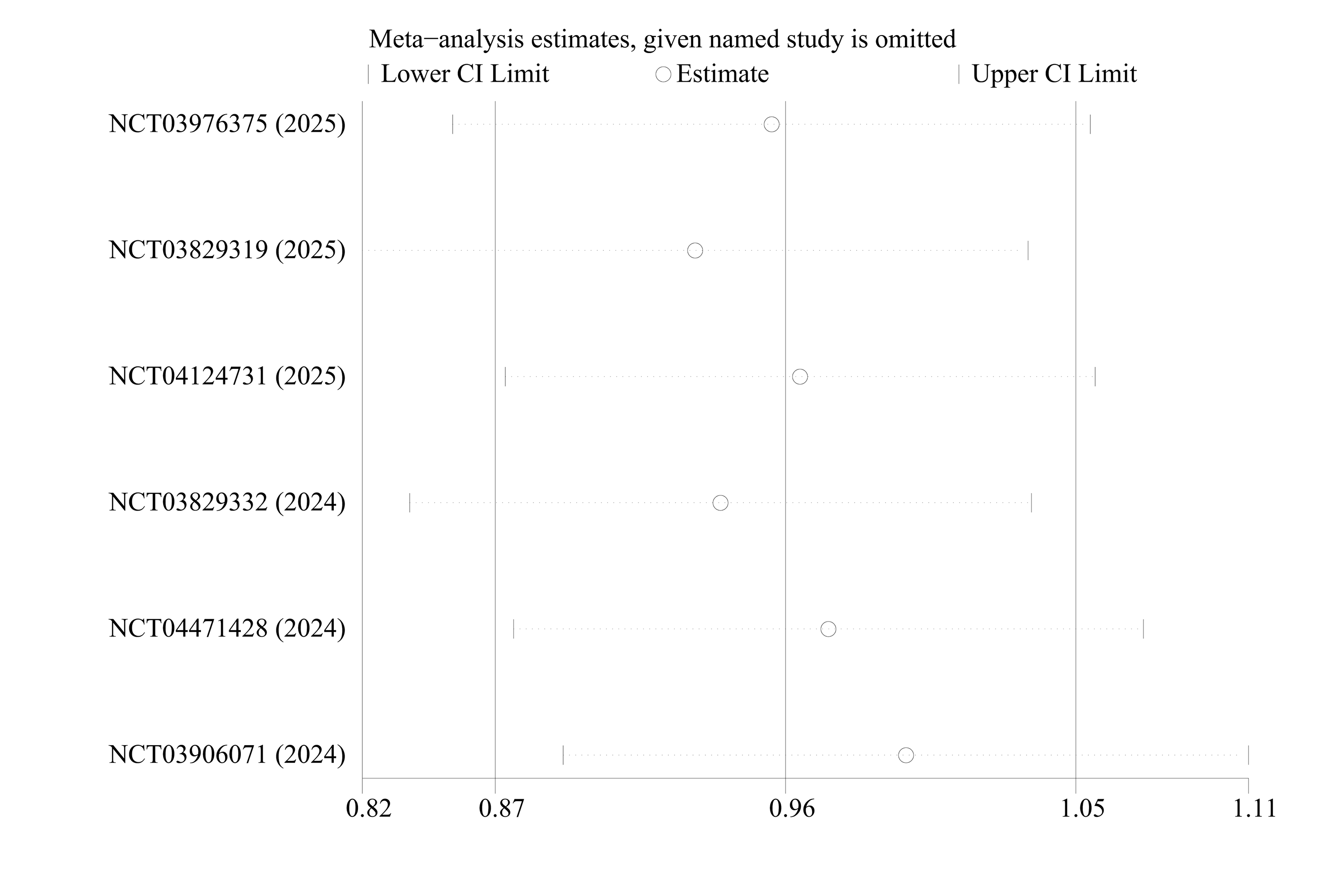

Supplement: Supplementary Figure 6 — Plot of the sensitivity analysis regarding the OS for the PD-1/PD-L1 inhibitors plus multi-targeted anti-angiogenic TKIs in advanced or metastatic NSCLC. [file Image6.tif]
