# Supplementary material for: Efficacy of PD-1/PD-L1 inhibitors combined with multi-targeted anti-angiogenic TKIs in advanced or metastatic NSCLC: A meta-analysis based on RCTs
Source: Front Oncol. 2026 Apr 1;16:1799126. doi: 10.3389/fonc.2026.1799126 (PMC13078995; doi:10.3389/fonc.2026.1799126)
Supplement: Supplementary file 7 [file DataSheet1.docx]

**Supplementary Table 1. Characteristics of the randomized controlled trials included in this meta-analysis.**

| Clinical trial  number | Year | Country | Recruitment time | Histology |
| --- | --- | --- | --- | --- |
| NCT03976375 | 2025 | Multi-countries | 2019.06-2022.02 | NSCLC |
| NCT03829319 | 2025 | Multi-countries | 2019.09-2021.03 | Nonsquamous  NSCLC |
| NCT04124731 | 2025 | China | 2019.11-2023.03 | NSCLC |
| NCT03829332 | 2024 | Multi-countries | 2019.04-2021.01 | NSCLC |
| NCT04471428 | 2024 | Multi-countries | 2020.10-2021.11 | NSCLC |
| NCT03906071 | 2024 | Multi-countries | NR | Nonsquamous  NSCLC |

NR, nor reported; NSCLC, non-small cell lung cancer.
